# ﻿A synopsis of the numbers of testicular follicles and ovarioles in true bugs (Heteroptera, Hemiptera) – sixty-five years of progress after J. Pendergrast’s review

**DOI:** 10.3897/zookeys.1136.96431

**Published:** 2022-12-19

**Authors:** Snejana Grozeva, Desislava Stoianova, Fedor Konstantinov, Nikolay Simov, Valentina G. Kuznetsova

**Affiliations:** 1 Institute of Biodiversity and Ecosystem Research, Bulgarian Academy of Sciences, Tsar Osvoboditel 1, Sofia, Bulgaria Institute of Biodiversity and Ecosystem Research, Bulgarian Academy of Sciences Sofia Bulgaria; 2 St. Petersburg State University, Universitetskaya nab. 7/9, St. Petersburg 199034, Russia St. Petersburg State University St. Petersburg Russia; 3 Zoological Institute, Russian Academy of Sciences, Universitetskaya emb. 1, St. Petersburg 199034, Russia Zoological Institute, Russian Academy of Sciences St. Petersburg Russia; 4 National Museum of Natural History, Bulgarian Academy of Sciences, Tsar Osvoboditel 1, Sofia, Bulgaria National Museum of Natural History, Bulgarian Academy of Sciences Sofia Bulgaria

**Keywords:** Accessory glands, ectadenia, evolution, mesadenia, ovarioles, phylogeny, testicular follicles, true bugs

## Abstract

The structure of testes and ovaries can be described in its simplest form by the number of follicles and ovarioles they contain. Sixty-five years after the last review of the internal reproductive systems in true bugs (Heteroptera), the data accumulated today on the number of testicular follicles and ovarioles in their gonads are summarized. In addition, data on the number and type (mesadenia/ectadenia) of accessory glands are given. The hemipteran suborder Heteroptera constitutes one of the most diverse groups of non-homometabolous (‘Hemimetabola’) insects, comprising more than 40,000 described species worldwide and approximately 100 families, classified into seven infraorders. Data are available for all infraorders; however, more than 90% of studied species belong to the largest and most evolutionarily derived infraorders Cimicomorpha and Pentatomomorpha. In true bugs, in general, the number of follicles varies from one to nine (in a testis), and the number of ovarioles varies from two to 24 (in an ovary). Seven follicles per testis and seven ovarioles per ovary prevail being found in approximately 43.5% (307 species) and 24.4% (367 species) of studied species, respectively. Such a structure of testes and ovaries is considered an ancestral character state in the Heteroptera. In the evolution of this group, the number of follicles and ovarioles both increased and decreased, but the trend towards a decrease clearly prevailed.

## ﻿Introduction

The hemipteran suborder Heteroptera (or true bugs) displays remarkable morphological variation and comprises more than 40,000 described species worldwide in ~ 100 families classified into seven infraorders including Enicocephalomorpha, Dipsocoromorpha, Nepomorpha, Gerromorpha, Leptopodomorpha, Cimicomorpha, and Pentatomomorpha. The majority of true bug species are herbivorous but some are predators and blood-suckers (Štys and Kerzhner 1975; Henry 2009; Schuh and Weirauch 2020).

The internal anatomy of the true bug males and females has been investigated for nearly two centuries. The first report on this topic appeared in the thirties of the 19^th^ century (Dufour 1833). Substantial comparative research on the internal anatomy of true bugs had accumulated by the 1960s mostly as a consequence of publications by J. Carayon, D. Leston, S. Miyamoto, T.E. Woodward, and J.G. Pendergrast (see Pendergrast 1957 and Miyamoto 1957 for references). Pendergrast (1957) published the first and, up to the present time, the only review of data available by that time on the internal reproductive organs of true bugs. Overall, Pendergrast summarized the literature (~ 90 titles) and his own data on ~ 50 families of Heteroptera and also shortly commented on the value of different parts of the male and female reproductive organs as characters in the taxonomy and classification of the Heteroptera. Despite the fact that Pendergrast singled out some organs as potentially promising for these purposes (in particular, testes in terms of the number of follicles they contain), he concluded that there is still very little data to make any generalizations. A huge contribution was made by Miyamoto (1957), who summarized the literature and own data on the structure of the ovaries in almost 300 representatives of ~ 40 families (some of them no longer families and other subfamilies recently erected to the separate families). Since the Pendergrast’s overview and Miyamoto’s list, ~ 120 original articles concerning the internal reproductive organs of Heteroptera have been published. In most cases, studies concern single species, and comparative data across multiple species of a group are few (e.g., Kumar 1967: Aradidae; Leston 1961a; Leston and Gibbs 1968; Malipatil 1978: Lygaeidae; Akingbohungbe 1983: Miridae; Grozeva and Kuznetsova 1992: Aradidae, Piesmatidae, Berytidae, Lygaeidae, Pyrrhocoridae). Much valuable information on true bugs can be found in special monographic works devoted to the reproductive systems of insects in general (Matsuda 1976; Büning 1994a, b; Chapman et al. 2013; Klowden 2013).

Although there is considerable diversity in detail, the internal parts of the male and female reproductive systems are organized similarly in different insects. In males of true bugs, it is formed by a pair of testes consisting of a variable number of testicular follicles, two different ducts, a median ejaculatory duct, and accessory glands. Accessory glands may be ectodermal or mesodermal in origin being known as ectadenia or mesadenia, respectively. Ectadenia open into the ejaculatory duct, whereas mesadenia open into the vasa deferentia or the distal end of the ejaculatory duct. In some species, both ectadenia and mesadenia are present, while other species have no accessory glands at all (Pendergrast 1957; Suppl. material 1). In females, the reproductive system is formed by a pair of ovaries consisting of a variable number of ovarioles, two lateral oviducts, a median common oviduct, and a spermatheca. The ovarioles are known to be of the meroistic telotrophic type (Chapman et al. 2013). In some species, accessory glands may also be present.

**Figure 1.**
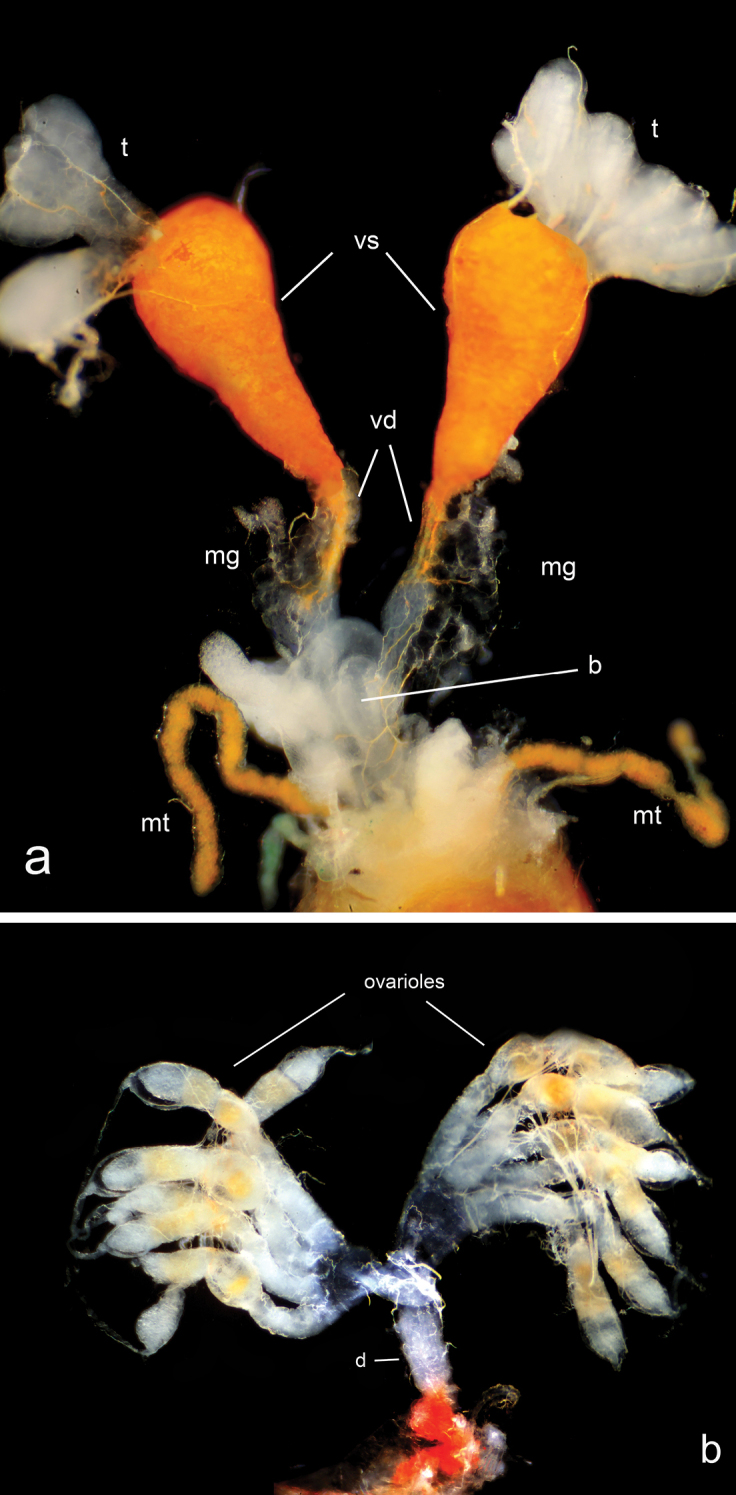
**a** Male (seven follicles) and **b** female (seven ovarioles) reproductive systems of *Arocatuslongiceps* Stål, 1872 (Lygaeidae). Abbreviations: t – testis, vd – vas deferens, vs – vesicula seminalis, mg – mesadenia, b – bulbus, mt – malpighian tubule, d – ductus (spermatheca and accessory glands were not visible in females).

**Figure 2.**
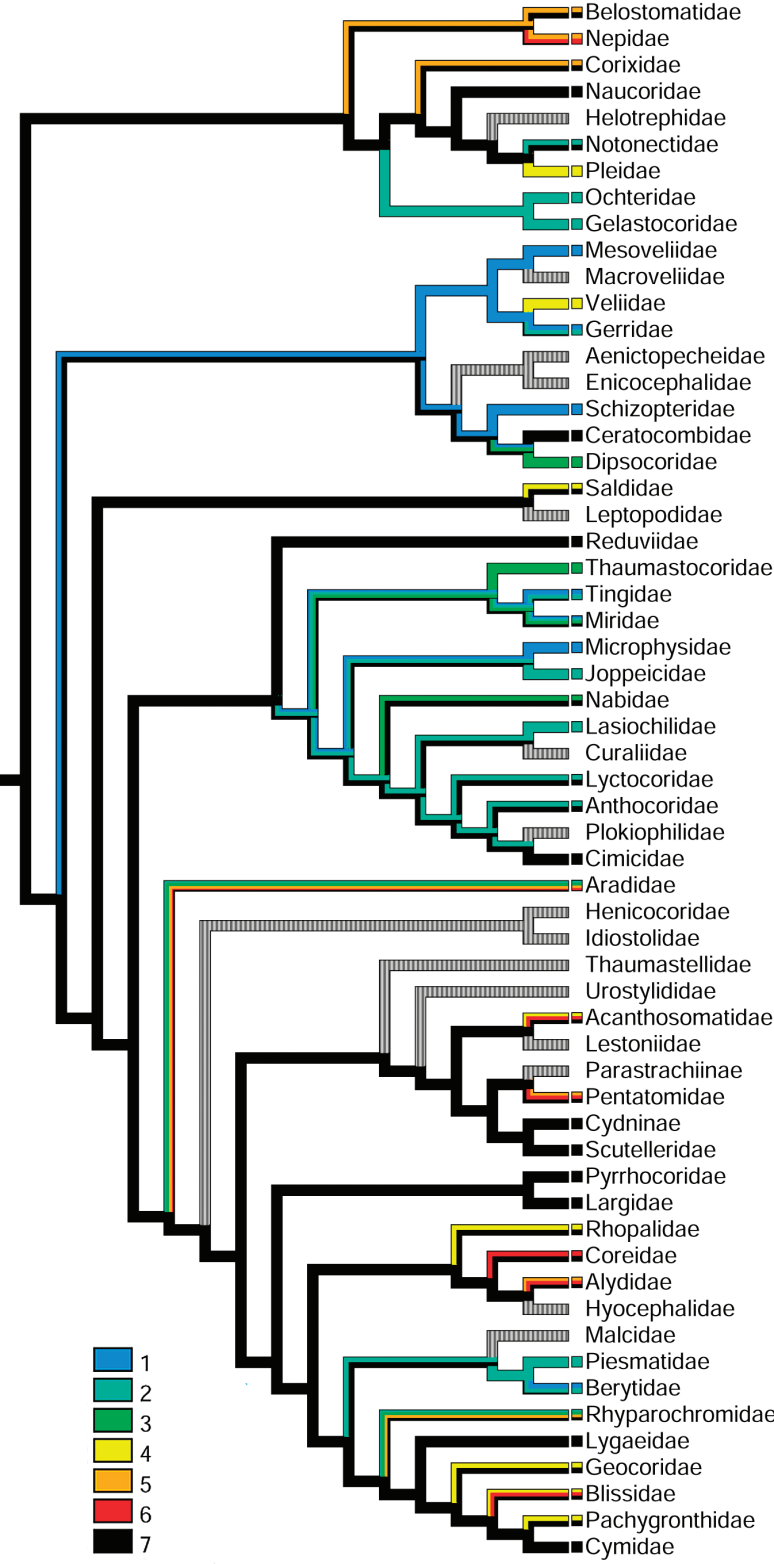
Testicular follicle numbers mapped on the true bug phylogeny after Weirauch et al. (2019).

**Figure 3.**
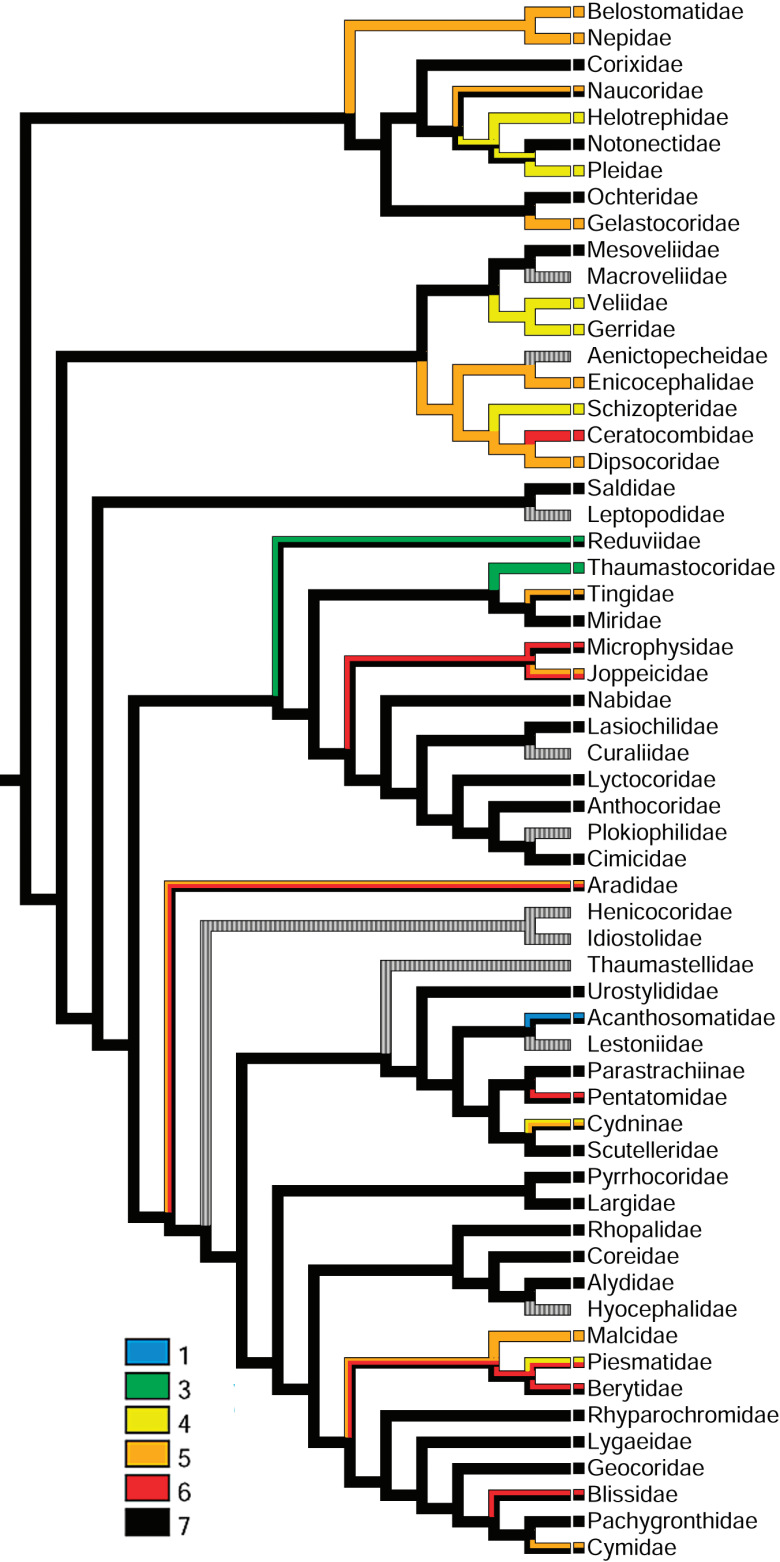
Ovariole numbers mapped on the true bug phylogeny after Weirauch et al. (2019).

During the years that have passed since the aforementioned overviews, the number of species and higher taxa of true bugs studied in relation to testes and ovaries has almost tripled, and it seems appropriate to publish an updated list. In this review article, all the data available today, including new data on 140 species obtained by the authors, are summarized in two tables. Suppl. material 1 includes all species studied to date in terms of the number of follicles and ovarioles. In some cases, additional information, e.g., on the number and type of accessory glands (ectadenia or mesadenia) in the male reproductive system, is also provided. Table 1 summarizes data presented in Suppl. material 1 and shows the variability of the number of follicles and ovarioles at different taxonomic levels, together with the modal values for each of the higher taxa explored. On Fig. 1, the internal reproductive organs of a male and a female of *Arocatuslongiceps* Stål, 1872 (Lydaeidae) is given. In Figs 2 and 3, character states of testicular follicle and ovariole numbers, respectively, are mapped onto the phylogenetic tree of Heteroptera families taken from Weirauch et al. (2019). In the final section of the review, the diversity of the analyzed characteristics and main tendencies of their evolution in true bugs are discussed. We hope that our review will be of importance for future work on the diversity of reproductive morphology within the Hemiptera, and that the data may provide additional information for understanding relationships between the higher taxa of true bugs.

**Table 1.** Distribution of numbers of follicles and ovarioles by families (Summarized based on Suppl. material 1).

Infraorder	Family	Subfamily (Tribe)	The number of species with a certain number of follicles per testis									The number of species with a certain number of ovarioles per ovary								
			1	2	3	4	5	6	7	8	9	2	3	4	5	6	7	8	17	24
**Cimicomorpha**	**Anthocoridae**			**20**		**1**			**3**								**6**			
Cimicomorpha	Anthocoridae	Almeidini							1								1			
Cimicomorpha	Anthocoridae	Anthocorini		10		1											1			
Cimicomorpha	Anthocoridae	Blaptopstethini		1																
Cimicomorpha	Anthocoridae	Cardiastethini		6													1			
Cimicomorpha	Anthocoridae	Oriini		1													2			
Cimicomorpha	Anthocoridae	Scolopini		2																
Cimicomorpha	Anthocoridae	Xylocorini							2								1			
**Cimicomorpha**	**Cimicidae**	**Cimicinae**							**2**								**4**			
**Cimicomorpha**	**Joppeicidae**			**1**											**1**					
**Cimicomorpha**	**Lasiochilidae**			**2**													**1**			
**Cimicomorpha**	**Lyctocoridae**			**3**													**4**			
**Cimicomorpha**	**Microphysidae**		**1**																	
**Cimicomorpha**	**Miridae**	**23 tribes**	**26**	**60**	**58**	**1**		**3**	**72**	**10**			**1**	**1**			**36**	**7**		
Cimicomorpha	Miridae	Bryocorinae (Bryocorini)	3	2																
Cimicomorpha	Miridae	Bryocorinae (Dicyphini)	14	2													12			
Cimicomorpha	Miridae	Bryocorinae (Eccritotarsini)															1			
Cimicomorpha	Miridae	Bryocorinae (Felisacini)	2																	
Cimicomorpha	Miridae	Bryocorinae (Monaloniini)		2	3															
Cimicomorpha	Miridae	Cylapinae (Fulviini)	1						1											
Cimicomorpha	Miridae	Deraeocorinae (Deraeocorini)	2	3					2	7								1		
Cimicomorpha	Miridae	Deraeocorinae (Hyaliodini)		2																
Cimicomorpha	Miridae	Isometopinae (Isometopini)															1			
Cimicomorpha	Miridae	Mirinae (Hyalopeplini)															1			
Cimicomorpha	Miridae	Mirinae (Mirini)		1				1	61	3							12	3		
Cimicomorpha	Miridae	Mirinae (Stenodemini)	1	1	4			2	6								5	3		
Cimicomorpha	Miridae	Orthotylinae (Ceratocapsini)		4																
Cimicomorpha	Miridae	Orthotylinae (Coridromiini)	1																	
Cimicomorpha	Miridae	Orthotylinae (Halticini)	1	2	5												1			
Cimicomorpha	Miridae	Orthotylinae (Nichomachini)			1															
Cimicomorpha	Miridae	Orthotylinae (Orthotylini)	1	40									1				2			
Cimicomorpha	Miridae	Phylinae (Cremnorrhini)			5				1											
Cimicomorpha	Miridae	Phylinae (Hallodapini)			1				1											
Cimicomorpha	Miridae	Phylinae (Nasocorini)			6															
Cimicomorpha	Miridae	Phylinae (Phylini)			28	1								1						
Cimicomorpha	Miridae	Phylinae (Pilophorini)		1	4												1			
Cimicomorpha	Miridae	Phylinae (Semiini)			1															
**Cimicomorpha**	**Nabidae**	**5 tribes**			**1**				**16**			**1**					**19**			
Cimicomorpha	Nabidae	Nabinae (Arachnocorini)			1												1			
Cimicomorpha	Nabidae	Nabinae (Gorpini)															1			
Cimicomorpha	Nabidae	Nabinae (Nabini)							12								10			
Cimicomorpha	Nabidae	Prostemmatinae (Phorticini)							1								1			
Cimicomorpha	Nabidae	Prostemmatinae (Prostemmatini)							3								4			
**Cimicomorpha**	**Polyctenidae**											**1**								
**Cimicomorpha**	**Reduviidae**	**12 subfamilies/11 tribes**		**2**	**1**		**1**		**74**	**1**	**1**		**2**			**1**	**57**	**5**		
Cimicomorpha	Reduviidae	Ectrichodiinae (Ectrichodiini)							2								4			
Cimicomorpha	Reduviidae	Ectrichodiinae (Tribelocephalini)							1											
Cimicomorpha	Reduviidae	Emesinae (Deliastini)							1											
Cimicomorpha	Reduviidae	Emesinae (Emesini)							4								3			
Cimicomorpha	Reduviidae	Emesinae (Leistarchini)							3											
Cimicomorpha	Reduviidae	Emesinae (Metapterini)					1		2								3	1		
Cimicomorpha	Reduviidae	Emesinae (Ploiariolini)		1	1				3							1				
Cimicomorpha	Reduviidae	Harpactorinae (Harpactorini)		1					19	1	1						20	4		
Cimicomorpha	Reduviidae	Harpactorinae (Rhaphidosomini)							3								2			
Cimicomorpha	Reduviidae	Holoptilinae											1							
Cimicomorpha	Reduviidae	Peiratinae							3								3			
Cimicomorpha	Reduviidae	Phymatinae							1				1							
Cimicomorpha	Reduviidae	Reduviinae							8								10			
Cimicomorpha	Reduviidae	Saicinae							1								1			
Cimicomorpha	Reduviidae	Salyavatinae							1								1			
Cimicomorpha	Reduviidae	Stenopodainae							3								8			
Cimicomorpha	Reduviidae	Triatominae (Rhodniini)							5								1			
Cimicomorpha	Reduviidae	Triatominae (Triatomini)							14											
**Cimicomorpha**	**Thaumastocoridae**				**2**							**1**	**3**							
Cimicomorpha	Thaumastocoridae	Thaumastocorinae			2							1	1							
Cimicomorpha	Thaumastocoridae	Xylastodorinae											2							
**Cimicomorpha**	**Tingidae**		**2**	**8**											**3**		**14**			
Cimicomorpha	Tingidae	Cantacaderinae	1	1											2					
Cimicomorpha	Tingidae	Tinginae	1	7											1		14			
Cimicomorpha	Tingidae	Vianaidinae																		
**Dipsocoromorpha**	**Ceratocombidae**	**Ceratocombinae**							**1**							**1**				
**Dipsocoromorpha**	**Dipsocoridae**				**4**										**3**					
**Dipsocoromorpha**	**Schizopteridae**	**Hysperosomatinae**	**2**											**1**						
**Enicocephalomorpha**	**Enicocephalidae**	**Enicocephalinae (Enicocephalini)**													**2**					
**Gerromorpha**	**Gerridae**		**1**	**6**										**15**						
Gerromorpha	Gerridae	Gerrinae		6										6						
Gerromorpha	Gerridae	Halobatinae												6						
Gerromorpha	Gerridae	Hermatobatinae												1						
Gerromorpha	Gerridae	Ptilomerinae												1						
Gerromorpha	Gerridae	Rhagadotarsinae												1						
Gerromorpha	Gerridae	Rheumatobatinae	1																	
**Gerromorpha**	**Hebridae**	**Hebrinae**		**2**											**3**					
**Gerromorpha**	**Hydrometridae**	**Hydrometrinae**															**4**			
**Gerromorpha**	**Mesoveliidae**	**Mesoveliinae**	**1**														**3**			
**Gerromorpha**	**Veliidae**		**4**									**1**		**10**						
Gerromorpha	Veliidae	Haloveliinae										1		1						
Gerromorpha	Veliidae	Microveliinae												8						
Gerromorpha	Veliidae	Rhagoveliinae	2											1						
Gerromorpha	Veliidae	Veliinae	2																	
**Leptopodomorpha**	**Aepophilidae**					1														
**Leptopodomorpha**	**Saldidae**	**Saldinae**				**1**			**2**								**2**			
**Nepomorpha**	**Aphelocheiridae**					**1**									**4**					
**Nepomorpha**	**Belostomatidae**						**6**		**1**						**5**					
Nepomorpha	Belostomatidae	Belostomatinae					5		1						3					
Nepomorpha	Belostomatidae	Lethocerinae					1								2					
**Nepomorpha**	**Corixidae**						**1**		**4**								**8**			
Nepomorpha	Corixidae	Corixinae							4								7			
Nepomorpha	Corixidae	Cymatinae					1										1			
**Nepomorpha**	**Gelastocoridae**			**3**											**1**					
Nepomorpha	Gelastocoridae	Gelastocorinae		2																
Nepomorpha	Gelastocoridae	Nerthrinae		1											1					
**Nepomorpha**	**Helotrephidae**	**Helotrephinae**												**1**						
**Nepomorpha**	**Micronectidae**			**3**													**1**			
**Nepomorpha**	**Naucoridae**								**3**											
Nepomorpha	Naucoridae	Limnocorinae							1											
Nepomorpha	Naucoridae	Naucorinae							2											
**Nepomorpha**	**Nepidae**						**2**	**1**						**1**	**6**					
Nepomorpha	Nepidae	Nepinae					1							1	2					
Nepomorpha	Nepidae	Ranatrinae					1	1							4					
**Nepomorpha**	**Notonectidae**			**1**					**2**								**4**			
Nepomorpha	Notonectidae	Anisopinae															1			
Nepomorpha	Notonectidae	Notonectinae		1					2								3			
**Nepomorpha**	**Ochteridae**			**2**													**7**			
**Nepomorpha**	**Pleidae**					**1**								**3**						
**Pentatomomorpha**	**Acanthosomatidae**	**Acanthosomatinae**				**1**		**1**	**3**								**7**		**4**	**1**
**Pentatomomorpha**	**Alydidae**						**1**	**1**	**3**								**6**			
Pentatomomorpha	Alydidae	Alydinae					1		3								4			
Pentatomomorpha	Alydidae	Micrelytrinae						1									2			
**Pentatomomorpha**	**Aradidae**	**6 subfamilies**		**1**	**2**	**1**	**18**	**3**				**1**	**4**	**2**	**19**	**6**	**1**			
Pentatomomorpha	Aradidae	Aneurinae					3	1							5					
Pentatomomorpha	Aradidae	Aradinae					1							1	2	3	1			
Pentatomomorpha	Aradidae	Calisiinae		1												1				
Pentatomomorpha	Aradidae	Carventinae			1	1	2						1	1	3					
Pentatomomorpha	Aradidae	Mezirinae			1		12					1	3		9					
Pentatomomorpha	Aradidae	Prosympiestinae						2								2				
**Pentatomomorpha**	**Artheneidae**	**Artheneinae**		**7**													**6**			
**Pentatomomorpha**	**Berytidae**	**3 subfamilies**	**6**	**5**												**1**	**6**			
Pentatomomorpha	Berytidae	Berytinae	2	5													2			
Pentatomomorpha	Berytidae	Gampsocorinae	2													1	1			
Pentatomomorpha	Berytidae	Metacanthinae	2														3			
**Pentatomomorpha**	**Blissidae**	**Blissinae**				**1**		**6**	**5**							**5**	**4**			
**Pentatomomorpha**	**Coreidae**	**2 subfamilies**						**1**	**16**								**17**			
Pentatomomorpha	Coreidae	Coreinae							16								16			
Pentatomomorpha	Coreidae	Pseudophloeinae						1									1			
**Pentatomomorpha**	**Cydnidae**	**7 subfamilies**							**4**					**1**	**1**		**12**			
Pentatomomorpha	Cydnidae	Cephalocteinae												1						
Pentatomomorpha	Cydnidae	Cydninae							2							1	6			
Pentatomomorpha	Cydnidae	Parastrachiinae															1			
Pentatomomorpha	Cydnidae	Sehirinae							2								4			
Pentatomomorpha	Cydnidae	Thyreocorinae															1			
**Pentatomomorpha**	**Cymidae**														**1**		**3**			
Pentatomomorpha	Cymidae	Cyminae													1		2			
Pentatomomorpha	Cymidae	Ontiscinae							**2**								1			
**Pentatomomorpha**	**Dinidoridae**								**1**								**2**			
Pentatomomorpha	Dinidoridae	Dinidorinae							1								1			
Pentatomomorpha	Dinidoridae	Megymeninae															1			
**Pentatomomorpha**	**Geocoridae**					**5**			**1**								**3**			
Pentatomomorpha	Geocoridae	Geocorinae				5											2			
Pentatomomorpha	Geocoridae	Henestarinae							1								1			
**Pentatomomorpha**	**Heterogasteridae**								**4**								**2**			
**Pentatomomorpha**	**Largidae**	**Physopeltinae**							**1**								**2**			
**Pentatomomorpha**	**Lygaeidae**			**1**					**12**								**14**			
Pentatomomorpha	Lygaeidae	Ischnorhynchinae							1								1			
Pentatomomorpha	Lygaeidae	Lygaeinae							6								6			
Pentatomomorpha	Lygaeidae	Orsillinae		1					5								6			
**Pentatomomorpha**	**Malcidae**	**Chauliopinae**													**1**					
**Pentatomomorpha**	**Ninidae**						**1**								**1**					
**Pentatomomorpha**	**Oxycarenidae**	**Oxycareninae**		**7**													**4**			
**Pentatomomorpha**	**Pachygronthidae**					**2**			**1**								**4**			
Pentatomomorpha	Pachygronthidae	Pachygronthinae															4			
Pentatomomorpha	Pachygronthidae	Teracriinae				2			1											
**Pentatomomorpha**	**Pentatomidae**	**6 subfamilies/18 tribes**			**5**	**8**	**7**	**26**	**36**	**1**			**1**	**1**		**12**	**43**			
Pentatomomorpha	Pentatomidae	Asopinae					1	2	4								9			
Pentatomomorpha	Pentatomidae	Discocephalinae							13	1										
Pentatomomorpha	Pentatomidae	Edessinae				3	1		1											
Pentatomomorpha	Pentatomidae	Pentatominae (Aeliini)						1	1							1				
Pentatomomorpha	Pentatomidae	Pentatominae (Agonoscelidini)						1									2			
Pentatomomorpha	Pentatomidae	Pentatominae (Antestiini)							1							1	1			
Pentatomomorpha	Pentatomidae	Pentatominae (Cappaeini)															2			
Pentatomomorpha	Pentatomidae	Pentatominae (Carpocorini)			1	2	1	12	4								6			
Pentatomomorpha	Pentatomidae	Pentatominae (Chlorocorini)						1	5											
Pentatomomorpha	Pentatomidae	Pentatominae (Eysarcorini)					1		1							5	4			
Pentatomomorpha	Pentatomidae	Pentatominae (Halyini)						1	2							1	1			
Pentatomomorpha	Pentatomidae	Pentatominae (Menidini)															1			
Pentatomomorpha	Pentatomidae	Pentatominae (Nezarini)			1			5	1								4			
Pentatomomorpha	Pentatomidae	Pentatominae (Pentatomini)			3	2		2	1								4			
Pentatomomorpha	Pentatomidae	Pentatominae (Piezodorini)					1	1									3			
Pentatomomorpha	Pentatomidae	Pentatominae (Sciocorini)															1			
Pentatomomorpha	Pentatomidae	Pentatominae (Sephelini)															1			
Pentatomomorpha	Pentatomidae	Pentatominae (Strachiini)					1		1							3				
Pentatomomorpha	Pentatomidae	Phyllocephalinae															1			
Pentatomomorpha	Pentatomidae	Podopinae (Graphosomatini)							1								2			
Pentatomomorpha	Pentatomidae	Podopinae (Podopini)					1						1	1			2			
Pentatomomorpha	Pentatomidae	Podopinae (Tarisini)				1										1				
**Pentatomomorpha**	**Piesmatidae**	**Piesmatinae**		**5**																
**Pentatomomorpha**	**Plataspidae**								**1**							**2**	**3**			
**Pentatomomorpha**	**Pyrrhocoridae**								**6**								**9**			
**Pentatomomorpha**	**Rhopalidae**					**4**	**1**		**5**								**7**			
Pentatomomorpha	Rhopalidae	Rhopalinae				2			3								6			
Pentatomomorpha	Rhopalidae	Serinethinae				2	1		2								1			
**Pentatomomorpha**	**Rhyparochromidae**	**6 tribes**		2	4		3		6								33			
Pentatomomorpha	Rhyparochromidae	Rhyparochrominae (Antillocorini)															2			
Pentatomomorpha	Rhyparochromidae	Rhyparochrominae (Drymini)		2													5			
Pentatomomorpha	Rhyparochromidae	Rhyparochrominae (Lethaeini)															5			
Pentatomomorpha	Rhyparochromidae	Rhyparochrominae (Myodochini)			4		3		1								12			
Pentatomomorpha	Rhyparochromidae	Rhyparochrominae (Rhyparochromini)							4								5			
Pentatomomorpha	Rhyparochromidae	Rhyparochrominae (Udeocorini)							1											
**Pentatomomorpha**	**Scutelleridae**								6								6			
Pentatomomorpha	Scutelleridae	Odontotarsinae							1								2			
Pentatomomorpha	Scutelleridae	Pachycorinae							1											
Pentatomomorpha	Scutelleridae	Scutellerinae							4								4			
**Pentatomomorpha**	**Stenocephalidae**								**1**								**1**			
**Pentatomomorpha**	**Termitaphididae**						**1**			**1**										
**Pentatomomorpha**	**Tessaratomidae**	**Natalicolinae**							**1**								**1**			
**Pentatomomorpha**	**Urostylididae**																**4**			
		**Total**	**43**	**141**	**77**	**25**	**42**	**43**	**294**	**12**	**1**	**5**	**11**	**36**	**50**	**28**	**367**	**12**	**4**	**1**

## ﻿Materials and methods

The current review article is part of a long-term research project dedicated to the study of true bugs, including the morphology of their reproductive system, cytogenetics, and evolution. The data we have obtained over years on the structure of testes and ovaries of true bugs in terms of the number of testicular follicles and ovarioles constitute at the present time a significant part of all such data currently available for the Heteroptera in general (Suppl. material 1). The material was collected during the expeditions of the authors (1986–2021) or provided by colleagues, mainly by D. Gapon, L. Hill, and H. Gunther. Material comes from different regions of Palaearctic (Europe: predominantly southern Europe and the Balkans, Central Asia, Caucasus) and some parts of the Old Tropics (Vietnam, Indonesia, Himalayas, India, Thailand, Tasmania). Species identification was made by authors or collectors (in case of donated material). Voucher samples are deposited in the insect collections of the National Museum of Natural History (**NMNHS**, Sofia, Bulgaria) and of the Institute of Biodiversity and Ecosystem Research (**IBER**, Sofia, Bulgaria) of the Bulgarian Academy of Sciences (**BAS**), and the Zoological Institute (**ZIN**) of the Russian Academy of Sciences (**RAS**), St. Petersburg, Russia.

Our original data cover 140 species belonging to 30 families, six infraorders, and represent 13.8% of all data on the structure of the testes and ovaries of true bugs accumulated to date and presented in Suppl. material 1. Some of these data have been published previously, the other part is presented here for the first time. Our study was carried out on specimens fixed in Carnoy fixative (3:1 96% ethanol and glacial acetic acid) that allowed both morphological and chromosomal analyses of the same individual. Material fixed in Duboscq Brasil (alcoholic Bouin’s) and ethanol (95%) was also used. The gonads were dissected out of the abdomen in a drop of 45% acetic acid on a microscope slide. The testicular follicles of the male and the ovarioles of the female were carefully separated from each other and counted under a stereomicroscope. Other parts of the reproductive system have also been studied, most notably, the accessory glands in males.

Classification used in this work follows Schuh and Weirauch (2020) except for Micronectidae which are treated as a separate family due to distinct cytogenetic features (Grozeva et al. 2008) and following Nieser (2002). All taxa are listed alphabetically.

Numbers of testicular follicles and ovarioles were treated as unordered states of two hidden characters and optimized on the Bayesian total-evidence tree of Heteroptera published by Weirauch et al. (2019) with all terminals of the original topology collapsed to family level. The phylogenetic reconstruction of Weirauch et al. (2019) was chosen for character mapping as the most recent analysis of the entire Heteroptera employing comprehensive coverage of true bug families. The data recorded in this publication comes from all available literature (Dufour 1833; Gross 1901; Marshall and Severin 1903; Bowen 1922a, b; Kerkis 1926; Ludwig 1926; Poisson 1927; Sarel-Whitfield 1929; Wille 1929; Hagan 1931; Payne 1934; Baumgartner and Witherspoon 1937; Kirkpatrick 1937; Larsen 1938; Slack 1938; Schneider 1940; Kullenberg 1947; Carayon 1949, 1950, 1951, 1954, 1955, 1972; Woodward 1949, 1950, 1977; Bonhag and Wick 1953; Davis 1955, 1956, 1966,1970; Pendergrast 1956, 1957; Miyamoto 1957, 1959; Scudder 1957; Barth 1958; Usinger and Matsuda 1959; Drake and Davis 1960; Schrader 1960a; Leston 1961a, b; Kumar 1962, 1964, 1965, 1967, 1969; Slater and Miyamoto 1963; Bhargava 1967; Leston and Gibbs 1968; Brunt 1971; Kauffman 1971; Huebner and Anderson 1972; Louis and Kumar 1973; Wightman 1973; Jansson and Scudder 1974; Malipatil 1978; Lung and Goeden 1982; Akingbohungbe 1983; Heming-van Battum and Heming 1986, 1989; Gonçalves et al. 1987; Ma and Ramaswamy 1987; Postle and Woodward 1988; Grozeva and Kuznetsova 1989, 1992; Socha et al. 1988; Papáček and Gelbič 1989; Biliński et al. 1990; Jawale and Ranade 1990; Gelbič et al. 1991; Hunt and Stebbing 1992; Papáček and Soldán 1992, 2008; Lent et al. 1994; Grozeva 1995, 2003, 2007; Grozeva and Nokkala 1996, 2001; Simiczyjew et al. 1996, 1998; Lalitha et al. 1997; Papáček et al. 1997; Štys et al. 1998; Simiczyjew 1999; Adams 2001; Couturier et al. 2002; Lis 2003; Kuznetsova et al. 2004, 2007; Lemos et al. 2005a, b, 2010; Santos et al. 2005; Grozeva et al. 2006, 2007, 2008, 2009, 2013; Jahnke et al. 2006; Kugler et al. 2006; Freitas et al. 2007a, b, 2008, 2010, 2012; Mróz 2007, 2012; Ogorzałek 2007; Pires et al. 2007; Grozeva and Simov 2008; Esquivel 2009; Marchini et al. 2009; Ogorzałek and Trochimczuk 2009; Souza et al. 2009; Chiang 2010, 2013; Van Doesburg et al. 2010; Horton and Lewis 2011; Mróz and Wojciechowski 2011; Souza et al. 2011, 2014; Uceli et al. 2011; Chiang et al. 2012; Kaur and Patial 2012, 2016; Soto et al. 2012; Gomes et al. 2013; Özyurt et al. 2013a, b, 2014a, b, 2015; Pluot-Sigwalt and Chérot 2013; Jyoti et al. 2015; Pereira et al. 2015; Cremonez et al. 2017, 2019; Elelimy et al. 2017a, b; Novais et al. 2017; Candan et al. 2018; Mahmood et al. 2018; Grodowitz et al. 2019, 2020; Khandelwal et al. 2019; Koçakoğlu et al. 2019; Xia 2019; Araújo et al. 2020a, b, 2021; Munhoz et al. 2020, 2021; Vélez et al. 2020; Gapon 2021; Oliveira et al. 2021; Bugaj-Nawrocka et al. 2022; Koutsogeorgiou et al. 2022; Samanta et al. 2022), and original observations (see Suppl. material 1).

The coding strategy aimed at reflecting diversity in numbers of follicles per testis (hereafter p.t.) and ovarioles per ovary (hereafter p.o.) observed within a given family. Therefore, states were coded as polymorphic in cases of large variability of follicle and ovariole numbers within a given family. This was the case for large and comparatively well studied families lacking widely accepted phylogenetic resolution e.g., Aradidae, Miridae, or Pentatomidae. However, in groups with a robust backbone phylogeny in place, e.g., Reduviidae (Weirauch 2008; Weirauch and Munro 2009; Hwang and Weirauch 2012), there was no doubt that the testis follicle number of seven is not only most common across the family, but may be treated as ancestral, and was coded as such. Within Veliidae, *Haloveliaseptentrionalis* Esaki, 1924 remains the only veliid species having two ovarioles p.o. Phylogeny of Gerromorpha by Damgaard (2008) rendered Haloveliinae as a sister group to Microveliinae which in turn forms a sister group to Gerridae. All veliid taxa including the second studied haloveliine species have four ovarioles p.o. and therefore we consider this number as an ancestral state for all veliids. History of both characters was traced using Mesquite 3.6.1 (Maddison and Maddison 2018) with the parsimony ancestral state reconstruction method (Figs 2, 3).

## ﻿Results and discussion

In this work, we have compiled ~ 1200 records of the numbers of testicular follicles and ovarioles in 1008 true bug species from across 63 families and seven infraorders, including Enicocephalomorpha (one family, two species), Dipsocoromorpha (three families, twelve species), Nepomorpha (eleven families, 50 species), Gerromorpha (five families, 50 species), Leptopodomorpha (two families, five species), Cimicomorpha (12 families, 474 species), and Pentatomomorpha (29 families, 437 species). Below we will comment on the available data for each infraorder, with emphasis on those families in which such data are more or less representative. Data on male accessory glands, their numbers, and origins (mesadenia/ectadenia), although provided in Suppl. material 1, are not specifically discussed since they are still very few.

### ﻿CIMICOMORPHA

With more than 20,000 species placed in 17 families, this highly diverse infraorder is the largest within Heteroptera (Weirauch and Schuh 2011). Members of the Cimicomorpha show a wide range of adaptations to different terrestrial habitats, highly diverse life histories and prey capture strategies, including predation and blood feeding in the Reduviidae, mostly plant feeding in the Miroidea, traumatic insemination in the Cimicoidea and Miridae, and ectoparasitism in the Cimicidae and Polyctenidae. Some cimicomorphans (Cimicidae, Triatominae in Reduviidae, and Polyctenidae) are of medical importance and hematophagous at all stages (Kerzhner 1981; Schuh and Štys 1991; Schuh and Slater 1995; Schuh 1995; Tatarnic et al. 2006; Schuh et al. 2009; Weirauch and Schuh 2011; Schuh and Weirauch 2020). Data on the number of follicles and/or ovarioles are currently available for 12 families and missing for Plokiophilidae, Velocipedidae, Pachynomidae, Curaliidae, and Medocostidae.

#### Anthocoridae (minute pirate bugs)

The family comprises more than 500 species with seven tribes (Péricart 1972; Schuh and Štys 1991; Péricart 1996; Jung et al. 2010; Schuh and Weirauch 2020). Data are available for the number of follicles in 29 species of 12 genera from all currently recognized tribes, although ~ 40% of studied species belong to the most species-rich tribe Anthocorini. In all but six species and in all but two (the Xylocorini and Almeidini) tribes, males have two follicles p.t. The exceptions are two of the eleven studied species of the genus *Anthocoris* Fallén, 1814 (Anthocorini) having four (*A.nemorum* (Linnaeus, 1761)) and even 5–7 (*A.bakeri* Poppius, 1913) follicles p.t. Four species, *Scoloposcelisobscurella* (Zetterstedt, 1838) (Scolopini), *Australmeidaderricki* (Gross, 1954), the single studied member of Almeidini, and the two studied species (subgenera *Proxylocoris* and *Arrostelus*) of the genus *Xylocoris* Dufour, 1931 in the monotypic tribe Xylocorini, have seven follicles p.t. The females of six studied species (in six genera of the Anthocorini, Almeidini, Cardiastethini, Oriini, and Xylocorini) have seven ovarioles p.o.

#### Cimicidae (bed bugs)

The family consists of ~ 110 species and 24 genera in six recognized subfamilies (Schuh and Štys 1991; Weirauch et al. 2019). The three studied species, all belonging to the genus *Cimex* Linnaeus, 1758 (Cimicinae), have seven follicles p.t. and seven ovarioles p.o.

#### Joppeicidae (joppeicid bugs)

The family contains only one species, *Joppeicusparadoxus* Putton, 1881. This relict family of non-specialized general predators feeding on small insects, has an obscure history of phylogenetic placement although sometimes regarded as related to the family Tingidae (Davis and Usinger 1970; Štys 1971). Thus, any information on this species is of considerable interest. Males of *J.paradoxus* have two follicles p.t., whereas females (according to different authors) have five or six ovarioles p.o. Five ovarioles are rarely observed in the Cimicomorpha (Davis and Usinger 1970) and its finding in Joppeicidae matches with *Cantacader* Amyot & Serville, 1843 species from the tingid subfamily Cantacaderinae and with *Corythuchamarmorata* (Uhler, 1878) from the other tingid subfamily Tinginae.

#### Lasiochilidae (lasiochilid bugs)

A small family of predaceous true bugs with general appearance like that of Anthocoridae and Lyctocoridae and formerly classified as a subfamily within the family Anthocoridae (Schuh and Štys 1991; Schuh and Slater 1995). In two species of the genera *Lasiochilus* Reuter, 1871 and *Plochiocorella* Poppius, 1909, males have two follicles p.t. The females of *Lasiochilus* sp. have seven ovarioles p.o.

#### Lyctocoridae (lyctocorid bugs)

A monotypic group of predominantly predaceous true bugs formerly classified, like the Lasiochilidae, as a subfamily within the family Anthocoridae (Schuh and Slater 1995). Lyctocoris (Lyctocoris) campestris (Fabricius, 1794) has two follicles p.t. and seven ovarioles p.o., and the same structure of the ovaries was found in *L.beneficus* (Hiura, 1957).

#### Miridae (plant bugs)

The variation of testis follicle number has been used as a potentially important character for the higher-level taxonomy and phylogeny of plant bugs by several authors including Leston (1961a, b), Akingbohungbe (1983), Grozeva and Simov (2008). In total, data on 259 species, less than 3% of the entire plant bug diversity, are currently available. Eight subfamilies and 39 tribes are generally recognized within Miridae (Cassis and Schuh 2012), and data on testis follicle numbers for many of these taxa are clearly insufficient due to sampling bias. Specifically, data on Psallopinae, Austromirini of Orthotylinae, Auricillocorini of Phylinae, three of six Deraeocorinae tribes, and four of six Mirinae tribes are lacking. Only a handful of species have been studied in the subfamilies Isometopinae (one) and Cylapinae (two), the deraeocorine tribe Hyaliodini (two), the phyline tribe Hallodapini (two), orthotyline tribes Coridromiini (one), and Nichomachini (one). However, available data for better studied taxa show a stable pattern of testis follicle numbers at the tribal and sometimes also subfamily levels. The modal numbers are briefly outlined below for each sufficiently studied subfamily.

##### Bryocorinae

In Dicyphini, a sister clade to the remaining bryocorines (Konstantinov et al. 2018), the presence of a single follicle p.t. was documented for all 21 examined species from four genera. The same appears to be true for the monotypic tribe Felisacini (two species examined). In two Dicyphini species, *Tupiocorisrhododendri* (Dolling, 1972) and Dicyphus (Brachyceroea) annulatus (Wolff, 1804), two follicles p.t. were found. The modal number of one was observed in the small tribe Bryocorini although Akingbohungbe (1983) documented two and two or three (“2-3”) follicles for *Monalocorisfilicis* (Linnaeus, 1758) and *M.americanus* Wagner & Slater, 1952, respectively. Seven examined species of Monaloniini and the only examined Eccritotarsini species *Stenopterocorislaticeps* China, 1944 have either one or three follicles p.t. Females of the permanently parthenogenetic species *Campyloneuravirgula* (Herrich-Schaeffer, 1835) (Eccritotarsini) were found to have seven ovarioles p.o.

##### Orthotylinae

Forty-three examined species of the largest tribe Orthotylini have two follicles p.t. Only *Cyrthorhinuscaricis* (Fallén, 1807) is an exception displaying testes with a single follicle each (Leston 1961a). In contrast, the tribe Halticini (eight species examined) shows no uniform pattern with a follicle number ranging from one to three.

##### Phylinae

Almost all species (28) of the large and diverse tribe Phylini have remarkably stable follicle number of three. Alternative structural variants found in several species, viz., *Oncotylusviridiflavus* (Goeze, 1778) (four-five), *Platyscytusdecempunctatus* (Carvalho, 1945) (four to six), and *Phylusmelanocephalus* (Linnaeus, 1767) (two or three in different testes of the only studied male) may be treated as isolated cases of specialization (Akingbohungbe 1983). The modal number for the tribe Pilophorini is also three, found in *Lasiolabopsobscurus* Poppius, 1914 and three species from the genus *Pilophorus* Hahn, 1926 (the fourth studied *Pilophorus* species, *P.cinnamopterus* (Kirschbaum, 1856) has two follicles p.t.). The same pattern applies for the tribes Cremnorrhini (with one exception of *Orectoderusobliquus* Uhler, 1876) and Nasocorini. The tribes Hallodapini and Semiini remain insufficiently studied, but also follow the pattern of three, as evidenced by the currently available data.

##### Deraeocorinae

Data on this subfamily are scarce and most examined deraeocorine species (17 of 19) belong to the tribe Deraeocorini. The testis follicle number ranges from one (*Zacheila* Odhiambo, 1961, *Fingulus* Distant, 1904, and *Finguluslibbyi* Akingbohungbe, 1981) to eight (most examined species of the subgenus Deraeocoris Kirschbaum, 1856) and no apparent pattern for this tribe could be established at this point.

##### Mirinae

Being the best studied plant bug tribe (73 species examined), Mirini unequivocally have seven testis follicles (in 66 species). The exceptions are very few and include *Garganusfusiformis* (Say, 1832) (two), *Capsodesgothicus* (Linnaeus, 1758) (six), *Neurocolpusjessiae* Knight, 1934 (eight), and *Poecilocapsuslineatus* (Fabricius, 1798) (eight). Noteworthy, in three species (*Adelphocorislineolatus* (Goeze, 1778), *Leptopternadolabrata* (Linnaeus, 1758), *Stenotusbinotatus* (Fabricius, 1794)) testis follicle number varies from seven to eight in different specimens or even in a single specimen of the same species. Stenodemini, the second well examined tribe (18 species), which forms a sister clade to all other tribes of the family (Schwartz 2008), also have the modal number of seven (in seven species). However, the tribe demonstrates exceptionally high diversity of testis follicle numbers, ranging from one (*Mimocepsinsignis* Uhler, 1890) to eight (*Leptopterna* spp.). Various authors documented contrastingly different numbers (3–7) for different species of the genus *Stenodema* Laporte, 1832.

Data on the number of ovarioles are available for a total of 48 species and 17 genera from the subfamilies Bryocorinae, Cylapinae, Deraeocorinae, Mirinae, Orthotylinae, Isometopinae, and Phylinae. Most species have seven ovarioles p.o. (found in 38 species, 24 genera, all subfamilies). Other numbers, including eight (in seven species, five genera), four (in one species), three (in one species), or 5–7 (in one species), occur sporadically in different subfamilies that have a modal number of seven.

#### Nabidae (damsel bugs)

A relatively small family of predaceous true bugs with more than 30 genera and ca. 400 species distributed in two subfamilies, Prostemmatinae (two tribes, each with only two genera) and Nabinae (four tribes) (Schuh and Štys 1991; Kerzhner 1996; Henry 2009). In Prostemmatinae, the four studied species representing the tribes Prostemmatini and Phorticini have seven follicles p.t. In the Nabinae, all ten species studied in the tribe Nabini (genera *Nabis* Latreille, 1802 and *Himacerus* Wolff, 1811) also have seven testis follicles. Available data suggest a remarkably stable follicle number of seven in these subfamilies. The only exception presently known in both Nabinae and Nabidae is *Arachnocoristrinitatis* Bergroth, 1916 (tribe Arachnocorini) which has three follicles p.t. but seven ovarioles p.o. All other studied species (16) from both subfamilies share the same pattern of seven ovarioles.

#### Polyctenidae (bat bugs)

The family currently comprises 32 species belonging to five genera and two subfamilies, Polycteninae and Hesperocteninae (Schuh and Weirauch 2020; Szentiványi et al. 2022). The females of the only studied species, *Hesperoctenesfumarius* (Westwood, 1874), have ovaries with two ovarioles each.

#### Reduviidae (assassin bugs)

With approximately 7000 species, 900 genera, and 25 subfamilies, Reduviidae are the second largest family of true bugs (Henry 2009; Hwang and Weirauch 2012; Weirauch et al. 2014). Data on the number of follicles and/or ovarioles are available for 111 species, 72 genera and 18 subfamilies. In most species, testes are composed of seven follicles each with a few exceptions. Particularly, three of the 16 studied Emesinae species differ from that pattern. The number of follicles in *Nesidiolestesroberti* Wygodzinsky, 1966 (Emesinae, Ploiariolini) is described as “fewer than 7”; however, a figure in the original paper (Wygodzinsky 1966: fig. 11J) shows a testis with three follicles. Males of *Saicellausingeri* Wygodzinsky, 1966 (Ploiariolini) and *Bobbavilliersi* Wygodzinsky, 1966 (Metapterini) were reported to have two and five follicles p.t., respectively. Within Harpactorinae (28 species studied), unusual testis follicle numbers were reported for *Polididusarmatissimus* Stål, 1859 with nine follicles p.t., *Nagusta* sp. (eight), and *Repiptataurus* (Fabricius, 1803) for which two follicles p.t. were tentatively given. In *P.armatissimus*, the structure of the testis was shown to be complex: the follicles form two groups, one consisting of seven long and wide follicles and the other consisting of two smaller follicles (Gapon et al. 2021).

The testis comprising seven follicles are suggested to be an ancestral trait for the Reduviidae (Gapon et al. 2021). This testis structure has been described for *Lisardavandenplasi* Schouteden, 1931 (Salyavatinae), eight species of the subfamily Reduviinae, 19 species of the Harpactorinae as well as for *Phymata* sp. from the Phymatinae complex, a sister group to the remaining “higher” reduviids. The number of ovarioles p.o. is seven in most species; however, some species have six (one species), three (two species), or eight (five species) ovarioles p.o. The available data suggest, thus, a high level of family stability in terms of both traits.

#### Thaumastocoridae (palm bugs)

The family comprises more than 30 species and six genera in two subfamilies, the Thaumastocorinae (21 species) and the Xylastodorinae (10 species) including nine recent and one described from the Dominican amber (Cassis et al. 1999; Schuh and Weirauch 2020). Data are available for four species and three genera of both subfamilies. Two species studied in the genus *Thaumastocoris* Kirkaldy, 1908 are similar in the number of follicles (two p.t.), but differ in the number of ovarioles p.o., which is three in *T.australicus* Kirkaldy, 1908 and two in *T.peregrinus* Carpintero & Dellapé, 2006. In Xylastodorinae, two species studied in the genera *Discocoris* Kormilev, 1955 and *Proxylastodoris* Heiss & Popov, 2002 are similar in having three ovarioles p.o.

#### Tingidae (lace bugs)

This family of herbivorous true bugs comprises ~ 2600 described species in more than 318 genera that are classified into the large subfamily Tinginae (~ 2500 species in 300 genera) and two smaller subfamilies, Cantacaderinae and Vianadinae (Golub et al. 2022). Data on follicle and/or ovariole numbers are available for a total of 25 species (12 genera) belonging to the Tinginae (21 species), Cantacaderinae (three), and Vianadinae (one). Within the Cantacaderinae, *Cantacaderquadricornis* (Lepeletier & Serville, 1828) and *C.quinquecostatus* (Fieber, 1844) were shown to have one and two follicles p.t., respectively. In the last of these species, females have ovaries with five ovarioles each, and the same structure of the ovaries is characteristic of *C.lethierryi* Scott, 1874. All studied species of the Tinginae (21 in ten genera) share consistent testis composition, with two follicles p.t. Fourteen species (ten genera) studied in terms of the ovaries have seven ovarioles; however, *Corythuchamarmorata* (Uhler, 1878) has five ovarioles p.o. The only studied species from the subfamily Vianaidinae, *Anommatocoriscoleopteratus* (Kormilev, 1955), has testes with a “bilobed follicle” each (Drake and Davis 1960). We are inclined to assume that this species actually has two follicles p.t.

#### Concluding remarks

In studied Cimicomorpha species, males may have 1–9 follicles p.t., and females may have 2–8 ovarioles p.o. Available data for the taxa where there are more data show a stable pattern of the number of follicles at different taxonomic levels. For example, seven follicles p.t. is the modal state in the families Reduviidae and Nabidae, in the tribes Mirini and Stenodemini (Miridae), and in the genus *Cimex* (Cimicidae); two follicles p.t. is the modal state in the family Anthocoridae, the subfamily Orthotylinae (Miridae) and the subfamily Tinginae (Tingidae); one follicle p.t. is the modal state in the tribe Dicyphini (Miridae). Data are scarcer on the ovariole numbers; however, in general, a pattern of seven ovarioles p.o. clearly predominates.

### ﻿DIPSOCOROMORPHA

The small infraorder Dipsocoromorpha or minute litter bugs comprises ~ 430 species from 70 genera that are classified into six morphologically distinct families (Knyshov et al. 2021). Data on the number of follicles and/or ovarioles are currently available for the families Dipsocoridae, Ceratocombidae, and Schizopteridae.

#### Ceratocombidae (litter bugs)

This small cosmopolitan family, historically treated as a subfamily of the Dipsocoridae and raised to family level by Štys (1970), comprises eight genera and ~ 50 species in two subfamilies (Slater 1982; Štys 1995b; Henry 2009). In Ceratocombinae, Ceratocombus (Ceratocombus) coleoptratus (Zetterstedt, 1819) displays seven follicles p.t., and C. (Xylonannus) sp. has six ovarioles p.o.

#### Dipsocoridae (jumping ground bugs)

This cosmopolitan family was recently redefined to contain three genera and ca.30 species with many more awaiting descriptions (Schuh and Weirauch 2020). Data on testis and/or ovary structure are available for seven species representing all three genera. In each species studied in genera *Cryptostemma* Herrich-Schaeffer, 1835 (two species), *Alpagut* Kıyak, 1995 (one), and *Pachycoleus* Fieber, 1860 (one), males have three follicles p.t. Females have five ovarioles p.o. in three studied *Cryptostemma* species while three ovarioles p.o. in *Pachycoleuspusillimus* (J. Sahlberg, 1870).

#### Schizopteridae (jumping soil bugs)

This family, which is the largest family of the infraorder, comprises approximately 355 species, 56 genera, and two subfamilies (Wygodzinsky 1955; Weirauch et al. 2018). Data are available for three representatives of the subfamily Hypselosomatinae. Males of *Hypselosomahickmani* Wygodzinsky, 1959 and *Pateenapolymitarior* Hill, 1980 have one follicle p.t., and females of *Hypselosoma* sp. have four ovarioles p.o.

#### Concluding remarks

In general, data are available for 12 species (seven genera), which is only 2.8% of the global diversity of the infraorder. Males of Dipsocoromorpha may have seven, three or one follicle p.t., the number three being characteristic of Dipsocoridae and the number one for Schizopteridae. Females may have four, five or six ovarioles p.o. It is worth noting the stability of the number of ovarioles in the genus *Cryptostemma* (three p.o.).

### ﻿ENICOCEPHALOMORPHA

Unique-headed bugs comprise only two small families, Aenictopecheidae (~ 20 described species, 11 genera and four subfamilies) and Enicocephalidae (~ 300 described species, 47 genera and five subfamilies) (Štys 1995a, 2002). Enicocephalomorpha have a checkered history of phylogenetic placement and were considered as a sister group to Dipsocoromorpha (Popov 1971), a sister group to all other Heteroptera (Štys and Kerzhner 1975; Wheeler et al. 1993; Xie et al. 2008), or rendered within a clade uniting Dipsocoromorpha and Gerromorpha (Johnson et al. 2018; Weirauch et al. 2019). Data on the structure of testes for aenictopecheids and enicocephalids are lacking. However, Štys and Baňař (2008) provided a drawing of the fusiform testis in the male of *Xenicocephalusjosifovi* Štys & Baňař, 2008 (Enicocephalidae, Enicocephalinae). Although the authors did not study the number of follicles, they described a testis as voluminous, suggesting that it consists of more than a single follicle. In two species of the Enicocephalidae, *Hoplitocorislewisi* Distant 1903 and *Stenopiratesjaponicus* (Esaki, 1935) (Enicocephalinae), females have five ovarioles p.o.

### ﻿GERROMORPHA

This infraorder of predatory, semiaquatic bugs, most of which live on the surface of the water or amongst floating plants, comprises more than 2100 species in 160 genera, eight families and five superfamilies (Polhemus and Polhemus 2008; Damgaard 2012). Data on the number of follicles and/or ovarioles are currently available for five families (not available for three very small families Hermatobatidae, Macroveliidae, and Paraphrynoveliidae).

#### Gerridae (water striders)

The family comprises at least 750 species and 71 genera in eight subfamilies and represents the second largest group of the infraorder in numbers of genera and species after the Veliidae (Schuh and Weirauch 2020). Data are available for the subfamilies Gerrinae (eight species, three genera), Halobatinae (six species, four genera), Hermatobatinae (one species), Ptilomerinae (one species), Rhagadotarsinae (two species, two genera), and Rheumatobatinae (one species). The number of follicles p.t. is two in all species of the Gerrinae, while males of *Rheumatobatescrassifemurcrassifemur* Esaki, 1926 (Rheumatobatinae) have one follicle p.t. Available data on the number of ovarioles demonstrate a highly stable pattern of four for all taxa (15 species, ten genera, five subfamilies without Rheumatobatinae for which there is no information).

#### Hebridae (velvet water bugs)

The family comprises 220 species and eight genera in two subfamilies (Andersen 1982; Schuh and Weirauch 2020). Data are available for the subfamily Hebrinae only, in which both studied species, Hebrus (Hebrusella) ruficeps Thomson, 1871 and H. (Hebrus) pusillus (Fallén, 1807), have two follicles p.t. These species and additionally H. (Hebrus) nipponicus Horváth, 1929 have five ovarioles p.o.

#### Hydrometridae (water measurers)

The family comprises at least 126 species and seven genera in three subfamilies (Andersen 1982). According to Pendergrast (1956), males of *Hydrometrastagnorum* (Linnaeus, 1758) (Hydrometrinae) have long and fusiform testes, each with supposedly one follicle. Females have seven ovarioles p.o. in each of the four studied species of the genus *Hydrometra* Latreille, 1797.

#### Mesoveliidae (pond treaders)

The family comprises ~ 50 species and 12 genera in two subfamilies and is considered a sister group to all other families of the infraorder (Schuh and Weirauch 2020). In the subfamily Mesoveliinae, *Mesoveliafurcata* Mulsant & Rey, 1852 has one follicle p.t. and seven ovarioles p.o. Two more species of this genus were reported to have the same structure of the ovaries.

#### Veliidae (riffle bugs)

The family comprises more than 970 species and 60 genera in six subfamilies and thus represents the largest family of the infraorder (Andersen 1982). Data are available for the subfamilies Haloveliinae (two species, two genera), Microveliinae (eight species, three genera), Rhagoveliinae (two species, two genera), and Veliinae (two species, two genera). All four riffle bug species studied in relation to the structure of the testes, two in the genus *Rhagovelia* Mayr, 1865 (Rhagoveliinae) and two others in the genus *Velia* Latreille, 1804 (Veliinae), have one follicle p.t. The number of ovarioles is also stable and equal to four in all studied species of the Microveliinae and in *Strongyloveliaformosa* Esaki, 1924 from the Haloveliinae, although another species of this subfamily, *Haloveliaseptentrionalis* Esaki, 1924, has two ovarioles p.o.

#### Concluding remarks

In 43 studied species (in 21 genera) of the infraorder Gerromorpha, males may have one or two follicles p.t., and females may have two, four, five, or seven ovarioles p.o. Available data for the taxa in which more data is available demonstrate a stable pattern of the follicle number (e.g., two in Gerridae and one in Veliidae) or of the ovariole number (e.g., four in Gerridae and Veliidae, and seven in Hydrometridae).

### ﻿LEPTOPODOMORPHA

The infraorder (shore bugs) comprises ca. 380 species in 42 genera and four extant families, including two larger Saldidae and Leptopodidae, both of worldwide distribution, and two rare and highly endemic families, the Omaniidae with four species in two genera, and monotypic Aepophilidae (Schuh and Polhemus 1980; Schuh et al. 1987; Schuh and Slater 1995; Polhemus and Polhemus 2012; Larivière and Larochelle 2019).

#### Aepophilidae (marine bugs)

This enigmatic taxon encompasses a single species, *Aepophilusbonnairei* Signoret, 1879 that has seven follicles p.t. in males.

#### Saldidae (shore bugs)

The family comprises ~ 335 species in 29 genera. Two studied species of the genus *Saldula* Van Duzee, 1914, *S.arenicola* (Scholtz, 1847) and *S.saltatoria* (Linnaeus, 1758), have seven follicles p.t., whereas *Halosaldalateralis* (Fallén, 1807) has four follicles p.t. Seven ovarioles p.o. were found in *Macrosaldulascotica* (Curtis, 1833) and *Saldulaarenicola* (Scholtz, 1847).

#### Concluding remarks

In five studied species of Leptopodomorpha (four genera, two families), males have seven or four follicles p.t. in four and one species, respectively. Note that the first number occurs in both explored families, and the same number of ovarioles p.o. is found in females of two studied species in two genera of shore bugs.

### ﻿NEPOMORPHA

The infraorder Nepomorpha or water bugs is one of the most specialized groups of heteropterans, with most of its species spending the entire life cycle within the water. It comprises more than 2300 species arranged in 140 genera and 13 families (Polhemus and Polhemus 2008; Ribeiro et al. 2018; Ye et al. 2019). Data on the number of follicles and/or ovarioles are currently available for all but two (Diaprepocoridae and Potamocoridae) families.

#### Aphelocheiridae (benthic water bugs)

The family comprises at least 78 species in the only genus *Aphelocheirus* Westwood, 1833 (Polhemus and Polhemus 2008). In *A.aestivalis* (Fabricius, 1794), males have four follicles p.t. and five ovarioles p.o., and the same structure of the ovaries was found in three more species of the genus.

#### Belostomatidae (giant water bugs)

The family comprises ca. 160 species and 11 genera in three subfamilies (Perez-Goodwyn 2006). Presently, data on testis and/or ovary structure are available for eight species in four genera of the subfamily Belostomatinae (*Belostoma* Latreille 1807, *Diplonychus* Laporte, 1833, *Appasus* Amyot & Serville, 1843, and *Adebus* Stål, 1862), and three species of the subfamily Lethocerinae (genus *Lethocerus* Mayr, 1853). All five studied *Belostoma* species have five follicles p.t., whereas the only studied *Diplonychus* species, *D.rusticus* (Fabricius, 1781), has seven follicles p.t. In *Lethocerusindicus* (Lepeletier & Serville, 1825), testes consist of five follicles each, and in *L.patruelis* (Stål, 1854), the number of follicles p.t. is questionable (five or four). Females of the four studied species in the genera *Belostoma* (one species), *Diplonychus* (one), *Adebus* (one), and *Lethocerus* (one) have five ovarioles p.o.

#### Corixidae (water boatmen)

The family comprises 607 species and 35 genera in four subfamilies (Polhemus and Polhemus 2008; Schuh and Weirauch 2020). Data on testis and/or ovary structure are available for seven species of the subfamily Corixinae and the only representative of the subfamily Cymatinae, *Cymatiacoleoptrata* (Fabricius, 1777). All studied Corixinae species from the genera *Sigara* Fabricius, 1775 (two species), *Cenocorixa* Hungerford, 1948 (one) and *Corixa* Geoffroy, 1762 (one) have seven follicles p.t., whereas male *C.coleoptrata* has five follicles p.t. The ovaries in all studied species (eight species, five genera, both subfamilies) consist of seven ovarioles each.

#### Gelastocoridae (toad bugs)

The family comprises at least 111 species and three genera from two subfamilies and belong to the secondarily terrestrial superfamily Ochteroidea (Hebsgaard et al. 2004). Data are available for four species. Two species in the genus *Gelastocoris* Kirkaldy, 1897 (Gelastocorinae) have two follicles p.t., and the same testis structure is typical for *Nerthraterrestris* (Kevan, 1948) from the subfamily Nerthrinae. Females of *Nerthramacrothorax* (Montrouzier, 1855) have five ovarioles p.o.

#### Helotrephidae (backswimmers)

The family comprises more than 170 species (Papáček and Zettel 2005) and is considered as a sister group to the Pleidae (Chen et al. 2005). *Helotrephesformosanus* Esaki & Miyamoto, 1943, the only explored species of the family, has four ovarioles p.o., that is the same number that have females in the Pleidae (see below).

#### Micronectidae (pygmy boatmen)

The family comprises 150 species and five genera in two subfamilies (Polhemus and Polhemus 2008; Schuh and Weirauch 2020). Data are available for three species of *Micronecta* Kirkaldy, 1897 (Micronectinae), in which males were reported to have two follicles p.t.

#### Naucoridae (creeping water bugs)

The family comprises ~ 420 species and 43 genera in six subfamilies (Sites 2022). All three species studied in the genera *Limnocoris* Stål, 1860 (Limnocorinae), *Pelocoris* Stål, 1876, and *Ilyocoris* Stål, 1861 (Naucorinae) have seven follicles p.t. Various authors reported different numbers of ovarioles (seven or five) p.o. for *Ilyocoriscimicoides* (Linnaeus, 1758).

#### Nepidae (water scorpions)

The family comprises 268 species and 15 genera from two subfamilies (Polhemus and Polhemus 2008; Schuh and Weirauch 2020). Data on the follicle number are available for three species in two genera, *Ranatra* Fabricius, 1790 (Ranatrinae) and *Nepa* Linnaeus, 1758 (Nepinae). *Nepacinerea* Linnaeus, 1758 have five follicles p.t., whereas males of *R.fusca* Palisot de Beauvois, 1820 and *R.linearis* (Linnaeus, 1758) have six and five follicles p.t., respectively. Among seven species with known structure of ovaries, all four *Ranatra* spp. share five ovarioles p.o., whereas two species of the genus *Laccotrephes* Stål, 1866 (Nepinae), *L.japonensis* Scott, 1874 and *L.maculatus* (Fabricius, 1775), have five and four ovarioles p.o., respectively.

#### Notonectidae (backswimmers)

The family comprises ~ 400 species and 11 genera in two subfamilies, Notonectinae and Anisopinae (Polhemus and Polhemus 2008; Schuh and Weirauch 2020). Data are available for four species only. In Notonectinae, *Notonectaglauca* Linnaeus, 1758 and *N.maculata* Fabricius, 1794 have seven follicles p.t., whereas *Martaregabentoi* Truxal, 1949, the only studied species in the Anisopinae, has two follicles p.t. Ovaries (examined in three species of *Notonecta* Linnaeus, 1758) consist of seven ovarioles each.

#### Ochteridae (velvety shore bugs)

The family comprises at least 68 species and belongs to the lineage of the aquatic Nepomorpha, which returned to a terrestrial way of life. Most authors consider Ochteridae as a sister group to the Gelastocoridae (e.g., Hebsgaard et al. 2004; Hua et al. 2009). The only examined species of the family, *Ochterusmarginatusmarginatus* (Latreille, 1804), shares two follicles p.t. with studied gelastocorids (see above), but has seven ovarioles p.o.

#### Pleidae (pygmy backswimmers)

The family comprises ~ 40 species in three genera (Polhemus and Polhemus 2008; Schuh and Weirauch 2020). Males of *Pleaminutissima* Leach, 1817 have four follicles p.t., and females have four ovarioles p.o. The same structure of the ovaries is characteristic of *Parapleaindistinguenda* (Matsumura, 1905) and *P.japonica* (Horváth, 1904).

#### Concluding remarks

In studied Nepomorpha species, males may have testes with different numbers of follicles (two to seven, except three), and females have ovaries with different numbers of ovarioles (four, five or seven). The numbers seven, five and two are most common, being found in 32%, 29% and 26% of studied species, respectively. In females, ovaries with five and seven ovarioles occur in 52% and 45% of studied species, respectively. Available data for relatively more fully studied taxa show a stable pattern of the follicle number at the generic level, e.g., five in *Belostoma* and two in *Micronecta* Kirkaldy, 1897, as well as at the subfamily level (seven in Corixinae), and it seems also at the family level (two in Gelastocoridae and Micronectidae). The ovariole numbers appear to show a stable pattern in some families (seven in Corixidae and Notonectidae, five in Aphelocheiridae, and four in Pleidae). However, only 50 species (in 25 genera) have been studied in the infraorder in general, i.e., ~ 2% of the global diversity of the group, which is, of course, too small to draw any conclusions.

### ﻿PENTATOMOMORPHA

With nearly 15 000 extant species, this worldwide distributed group is the second largest infraorder of true bugs, with 40 currently recognized families arranged in six superfamilies viz., Aradoidea, Idiostoloidea, Coreoidea, Lygaeoidea, Pyrrhocoroidea, and Pentatomoidea (Weirauch et al. 2019; Schuh and Weirauch 2020). The majority of pentatomorphan insects are terrestrial and phytophagous, but some of them are predaceous (Schuh and Slater 1995; Weirauch and Schuh 2011; Liu et al. 2019). Data on the number of follicles and/or ovarioles are currently available for 433 species (2.4%), 274 genera and 28 families (70%) in all superfamilies except Idiostoloidea, a small and poorly studied group containing six species only.

#### ARADOIDEA

##### Aradidae (flat bugs)

The family comprises approximately 1900 species, more than 230 genera, and eight subfamilies (Heiss 2001). Data on the number of follicles and/or ovarioles are available for 41 species, 17 genera and six subfamilies (unavailable for Isoderminae and Chinamyersiinae).

**Prosympiestinae.** In two studied species of the genus *Prosympiestus* Bergroth, 1894, males have six follicles p.t. and females have six ovarioles p.o.

**Carventinae.** Data are available for the genera *Carventus* Stål, 1865 (two species), *Euricoris* Kormilev, 1957 (two species), and *Paracarventus* Kormilev, 1964 (one species). Both testes and ovaries have the same range of numbers, three, four, or five, in each case the numbers of follicles and ovarioles coinciding in the same species.

**Calisiinae.** In *Calisiushackeri* Kormilev, 1958, males have two follicles p.t. and females have six ovarioles p.o.

**Aneurinae.** Data are available for the genera *Aneurus* Curtis, 1825 (six species) and *Paraneurus* Jacobs 1986 (one species). Both genera are characterized by a stable pattern of five follicles p.t. and five ovarioles p.o., with the only exception of A. (Aneurus) laevis (Fabricius, 1775) having six follicles p.t. For Aneurus (Aneurodes) avenius (Dufour, 1833) four and five ovarioles are reported by different authors.

#### COREOIDEA

##### Coreidae (leaf-footed bugs)

The family comprises more than 2570 extant species described in four subfamilies and 37 tribes (Coreoidea SF Team 2019). Data are available for the subfamilies Coreinae and Pseudophloeinae. The first is distinguished by the high stability of the structure of the testes and ovaries having seven follicles p.t. in all 16 studied species in 14 genera and the same number of ovarioles p.o. in all 16 studied species in nine genera. In the second subfamily, *Coriomerishirticornis* (Fabricius, 1794) also has seven ovarioles p.o., although *Ceraleptuslividus* Stein, 1858 has six follicles p.t.

##### Rhopalidae (scentless plant bugs)

The family comprises more than 200 species in 30 genera and two subfamilies, the Rhopalinae and the Serinethinae (Schuh and Weirauch 2020). Data are available for 11 species and seven genera of both subfamilies. A testis may consist of four, five, or seven follicles, but the number of seven prevails (found in seven species, six genera, both subfamilies).

##### Stenocephalidae (spurgebugs)

In the sole genus of the family, *Dicranocephalus* Hope, 1831, one species has seven follicles p.t. and seven ovarioles p.o. (*D.agilis* (Scopoli, 1763)), whereas another species (*D.albipes* (Fabricius, 1781)) has five follicles p.t.

#### LYGAEOIDEA

##### Artheneidae

This group contains slightly more than 20 species arranged in seven genera and three subfamilies, with most species belonging to Artheneinae, while Dilompinae and Nothochrominae remain monotypic (Henry 1997a; Schuh and Weirauch 2020). The reproductive system was studied in seven species belonging to three genera or one-third of the described diversity of Artheneinae. A uniform pattern of two follicles p.t. and seven ovarioles p.o. was revealed in all cases.

##### Berytidae (stilt bugs)

This worldwide family comprises ~ 37 genera and 174 species in three subfamilies (Henry 1997b, c; Henry et al. 2015). Data are available for ~ 19% and 8% of genera and species, respectively, belonging to all subfamilies, Berytinae (seven species, three genera), Gampsocorinae (three species, one genus), and Metacanthinae (four species, three genera). There is one follicle p.t. in the two latter subfamilies and two, but sometimes one, in the Berytinae. The number of ovarioles is seven p.o. in all but one species studied. These species represent all three subfamilies and the only exception is *Gampsocorisviridiventris* (Matsumura, 1907) (Gampsocorinae), which has six ovarioles p.o. although another species of the same genus, *G.culicinus* Seidenstücker, 1948, has seven.

##### Blissidae

This family of strongly flattened and sap sucking lygeoids has a worldwide distribution and currently contains more than 400 species belonging to ca. 50 genera (Schuh and Weirauch 2020). No suprageneric classification was ever proposed for this group, and the reproductive system was studied in 14 species from six genera. Of these, the most frequent number of follicles p.t. equals six, documented in all studied *Blissus* Burmeister, 1835 spp., *Macropesraja* Distant, 1909 and *Iphicratesspinicaput* (Scott, 1874). However, seven follicles p.t. were also frequently observed across some studied taxa, e.g., *Ischnodemus* spp., *Macropesobnubilus* (Distant, 1883), *Iphicratesspathus* Slater, 1961, and *Heinsiusexplicatus* Distant, 1901, and four follicles were documented for *Caveleriussaccharivorus* (Okajima, 1922). The number of ovarioles is either seven or six and appears to correspond to the number of follicles in males of a given species. *Caveleriussaccharivorus* (Okajima, 1922) forms the only exception, having six ovarioles but four follicles.

##### Cymidae

This group contains at least 64 species in ten genera classified into two subfamilies, Cyminae and Ontiscinae (Schuh and Weirauch 2020). Despite easy accessibility for specimen collecting, data on the male reproductive system are scarce. Two species of the genus *Ontiscus* Stål, 1874 were studied so far and possess both seven follicles p.t. Seven ovarioles p.o. were documented for three species from both subfamilies, and five ovarioles p.o. were registered in *Cymusaurescens* Distant, 1883.

##### Geocoridae (big-eyed bugs)

This family comprises 27 genera and ~ 280 species in five subfamilies (Henry et al. 2015). In the genus *Geocoris* Fallén, 1814 (Geocorinae), males have four follicles p.t. (five species) and females have seven ovarioles p.o. (two species). Males of *Henestarishalophilus* (Burmeister, 1835) (Henestarinae) have seven follicles p.t. and females have seven ovarioles p.o.

##### Heterogastridae

This small family currently consists of 23 genera and at least 105 species (Schuh and Weirauch 2020), four of which were studied in regard to the structure of the testes and ovaries. All three studied species of the genus *Heterogaster* Schilling, 1829 and *Platyplaxsalviae* (Schilling, 1829) have seven follicles p.t., and females of two studied *Heterogaster* spp. have seven ovarioles p.o.

##### Lygaeidae (seed bugs)

The family comprises 107 genera and more than 970 species in three subfamilies (Henry et al. 2015). Data are available for 16 species and six genera of the subfamilies Ischnorhynchinae (two species, two genera), Lygaeinae (nine species, five genera), and Orsillinae (seven species, three genera). Except for *Paranysiusfraterculus* (Burmeister, 1835) (Orsillinae) having two follicles p.t., the number of follicles and ovarioles in seed bugs equals seven (p.t. and p.o., respectively).

##### Malcidae

The family comprises more than 40 described species and three genera in two subfamilies (Schuh and Weirauch 2020). In the only studied species, *Chauliopsfallax* Scott, 1874 (Chauliopinae), females have five ovarioles p.o.

##### Ninidae

Five genera and 14 species are recognized in this small pantropical group (Schuh and Weirauch 2020). Among the two species studied, males of *Ninomimusflavipes* (Matsumura, 1913) have five follicles p.t., and the females of *Ninusinsignis* Stål, 1860 have five ovarioles p.o.

##### Oxycarenidae

This worldwide distributed family contains slightly less than 150 species from 27 genera not arranged into subfamilies or tribes (Schuh and Weirauch 2020). All studied species have two follicles p.t. (eight species from six genera) and seven ovarioles p.o. (four species from four genera).

##### Pachygronthidae

With more than 80 species arranged in 14 genera, this predominantly tropical and subtropical taxon clusters into two subfamilies, Pachygrontinae and Teracriinae (Slater 1955). Available data on the morphology of the reproductive system are scarce, with four (two species) and seven (one species) follicles p.t. documented for Teracriinae. Female reproductive system has only been studied in four *Pachygrontha* spp. (Pachygronthinae) resulting in seven ovarioles p.o. in each case.

##### Piesmatidae

This small but nonetheless worldwide distributed group contains less than 50 species arranged in six genera and two morphologically and ecologically distinct subfamilies, Piesmatinae and Psamminae (Henry 1997a; Schuh and Weirauch 2020). While the reproductive system of Psamminae has never been studied, all six species from two genera of the subfamily Piesmatinae were shown to have two follicles p.t. The ovaries have been studied in only one species, *Parapiesmaquadratum* (Fieber, 1844), that has been observed to have four or six ovarioles p.o.

##### Rhyparochromidae (dirt-colored seed bugs)

The family comprises more than 2000 species in two subfamilies, Plinthisinae and Rhyparochrominae, the latter with 14 tribes (Henry 1997a). Data are available for 40 species and 29 genera of the Rhyparochrominae (six tribes). The number of ovarioles p.o. is seven in all studied species, whereas the number of follicles p.t. is either two (in Antillocorini and Drymini), three or five (in different genera of Myodochini), or seven (in the single species of Myodochini, and in all studied species of Udeocorini and Rhyparochromini), seven clearly prevailing in the family.

#### PENTATOMOIDEA

##### Acanthosomatidae

The family comprises more than 50 genera and more than 280 species in three subfamilies (Schuh and Slater 1995; Henry 2009; Schuh and Weirauch 2020). Data on the testis and ovary structure are available for 15 species within five genera of the subfamily Acanthosomatinae. The number of follicles may be seven (three species, three genera), four (one species) or six (one species), and both last numbers occur in the genus *Rhopalimorpha* Dallas, 1851 (*Rh.lineolaris* Pendergrast, 1950 and *Rh.obscura* Dallas, 1851, respectively). The number of ovarioles is seven in the majority (seven) of species. The genus *Elasmucha* Stål, 1864 is of interest showing seven ovarioles in *E.graminea* (Distant, 1883) while unusually high numbers, 17 and even 24, in five other species.

##### Cydnidae (burrowing bugs)

The family comprises more than 1180 species in ca. 145 genera worldwide, arranged in nine subfamilies (Henry 2009). Data are available for the subfamilies Cephalocteinae, Cydninae, Parastrachiinae, Sehirinae, and Thyreocorinae. In all species studied in the subfamilies Cydninae (two species, two genera) and Sehirinae (two species, two genera), testes consist each of seven follicles. Fourteen species (13 genera, five subfamilies) were studied with respect to the ovaries, and twelve of them have seven ovarioles p.o.; however, *Stibaropus* sp. (Cephalocteinae) and *Chilocorisconfusus* Horváth, 1919 (Cydninae) have four and five ovarioles p.o., respectively.

##### Dinidoridae

The family comprises ~ 100 species in 16 genera and two subfamilies (see Lis et al. 2012 for references). The testis and ovary of *Coridiusjanus* (Fabricius, 1775) (Dinidorinae) consist of seven follicles and seven ovarioles, respectively, and the same structure of the ovaries is found in *Megynemumgracilicorne* Dallas, 1851 (Megymeninae).

##### Pentatomidae (stink bugs)

This third largest true bug family comprises almost 5 000 species in ca. 940 genera distributed in nine subfamilies including Asopinae, Cyrtocorinae, Discocephalinae, Edessinae, Pentatominae, Phyllocephalinae, Podopinae,Serbaninae, and Stirotarsinae (Rider 2006; Rider et al. 2018; Schuh and Weirauch 2020). Data on the testes and ovaries are available for all subfamilies, except Cyrtocorinae, Serbaninae, and Stirotarsinae. In total, the testis structure was studied in males of 88 species of 63 genera and five subfamilies (there is no data for Phyllocephalinae), and the structure of ovaries is studied in females of 57 species in 41 genera (Pentatominae, Asopinae, Podopinae, and Phyllocephalinae). The number of follicles varies from three to eight, but seven follicles appear to be the most frequent state being found in each subfamily and in most studied species and genera. The observed numbers of ovarioles were three, four, six, or seven and, like in the case of the number of follicles, seven ovarioles p.o. is the most common pattern found in more than two thirds of species and genera studied in this regard (43 species, 36 genera, all four subfamilies). Other numbers were observed in 14 species only. Nine of these species belong to the genera *Eysarcoris* Hahn, 1834 and *Eurydema* Laporte de Castelnau, 1833 (Pentatominae, Eysarcorini, and Strachiini respectively), for which the characteristic number is six. For some species, different authors reported different numbers for the same species, for example, six and seven for *Eysarcorisventralis* (Westwood, 1837) and *Nezaraviridula* (Linnaeus, 1758). In Bagrada (Bagrada) hilaris (Burmeister, 1835), different numbers of ovarioles were observed in different ovaries of the same specimen.

Pentatomids are remarkable in that their testes may possess a so-called “harlequin” lobe, in which meiosis is aberrant leading to the production of spermatids carrying numerically unbalanced chromosome complement with an abnormal and highly variable chromosome number (Schrader 1960a). Speculation on the evolutionary aspects of the harlequin lobe can be found in Schrader (1960b). To date, this specific follicle has been found mostly in the subfamilies Discocephalinae, Edessinae, and Pentatominae (Rebagliati et al. 2005).

##### Plataspidae

The family comprises ~ 530 species in 56 genera (Jessop 1983). The only species studied with respect to the testes, *Coptosoma* sp., has seven follicles p.t., and five species studied with respect to the ovaries have seven (three species, two genera) and six (two species, two genera) ovarioles p.o.

##### Scutelleridae (jewel bugs)

The family comprises ~ 80 genera and 500 described species worldwide (Tsai et al. 2011). Data are available for 11 species in nine genera of the subfamilies Odontotarsinae (three species, two genera), Pachycorinae (one species), and Scutellerinae (seven species, six genera). All species have seven follicles p.t. (six species studied) and seven ovarioles p.o. (six species studied).

##### Tessaratomidae

The family comprises three subfamilies, 49 genera and ~ 235 species worldwide (Rolston et al. 1993). The only studied species, *Cyclogastrideanigromarginalis* Reuter, 1884 (Natalicolinae), has seven follicles p.t. and seven ovarioles p.o.

##### Urostylididae

The family comprises four genera and ca. 80 species (Rider et al. 2018). No data on the male reproductive system are available at the moment. In three species of the genus *Urostylis* Westwood, 1837 and in *Urochelaluteovaria* Distant, 1881, females have seven ovarioles p.o.

#### PYRROCOROIDEA

##### Largidae (bordered plant bugs)

The family comprises ~ 200 species in two subfamilies worldwide. In the subfamily Physopeltinae, *Physopeltagutta* (Burmeister, 1834) has seven follicles p.t. and seven ovarioles p.o.

##### Pyrrhocoridae (cotton stainers)

The family comprises more than 300 species worldwide in at least 30 genera and two subfamilies. Data are available for 11 species in four genera of the subfamily Pyrrhocorinae; all examined species have seven follicles p.t. and seven ovarioles p.o. The only exception is Dysdercus (Paradysdercus) koenigii (Fabricius, 1775) with a variable number of 5–7 follicles p.t.

##### Concluding remarks

In Pentatomomorpha species studied, males may have 3–8 follicles p.t., and females 3–7 ovarioles p.o., except the genus *Elasmucha* (Acanthosomatidae) in which a unique diversity (7–24 ovarioles p.o.) has been reported for the five studied species. The number seven is characteristic for some higher taxa studied in relation to the structure of the testes and ovaries (among the better studied families, these are Coreidae, Cydnidae, Lygaeidae, and Pentatomidae). The exception is the family Aradidae for which this number seems to not be characteristic of either the ovaries or the testes, with seven being only reported for the ovaries of Aradus (Aradus) pictus Baerensprung, 1859. In some families (e.g., Artheneidae, Oxycarenidae, Berytidae), the number seven is typical for the ovaries but not for the testes, which consist either of two follicles (in the first two families) or of one follicle (in the third family).

## ﻿Conclusions

In this study, we analyzed the number of follicles in testes of 705 species (420 genera, 58 families) and the number of ovarioles in ovaries of 504 species (334 genera, 61 families) across all seven major lineages of the suborder Heteroptera (Figs 2, 3, Suppl. material 1). Our comprehensive review showed that the gonads in true bugs are paired organs, and the testis and the ovary may consist of a variable number of follicles and ovarioles ranging from one to nine and from one to eight (while occasionally even to 24 in some Acanthosomatidae) respectively. Approximately 40% of species have seven follicles p.t. and ~ 68% of species have seven ovarioles p.o. Numbers exceeding this modal value are rare; the exception is eight follicles in the genus *Deraeocoris* Kirschbaum, 1855 (Miridae), in which almost half of the studied species have this number. Lower numbers, on the other hand, are quite common; moreover, sometimes they characterize taxa of high rank. In the families Miridae, Pentatomidae, and Reduviidae, for which there is relatively much data, a variety of numbers is observed. It is most pronounced in the Miridae in which data on testes and ovaries are available for 220 and 48 species, respectively. Although the number of ovarioles in most mirid species is seven, the number of follicles varies widely, from one to eight (except five), the number seven and lower numbers (1–3) occurring with almost equal frequency. Some other families, e.g., Anthocoridae, Aradidae, Belostomatidae, Berytidae, Gerridae, Oxycarenidae, and Tingidae, usually have low numbers of follicles. Noteworthy is also the family Aradidae, in which ovarioles vary in number, with numbers below the modal predominating. However, there is still very little data for all these families.

Ancestral reconstruction of testes suggests seven follicles p.t. as an ancestral state for true bugs (Fig. 2). This number was also recovered for the common ancestors of Pentatomomorpha and all its superfamilies except Aradoidea, Cimicomorpha, Leptopodomorpha, and Nepomorpha. This is consistent with the fact that seven follicles p.t. were documented in the majority of studied species across Heteroptera (Suppl. material 1; Akingbohungbe 1983). However, the common ancestor of Gerromorpha appears to have a testis with one follicle, while the parsimony reconstruction for Enicocephalomorpha and Dipsocoromorpha indicates equal probability of one or seven follicles as an ancestral state. This variation in ancestral traits is noteworthy, given the ongoing debates on the backbone phylogeny of the Heteroptera, implying Enicocephalomorpha (Wheeler et al. 1993; Xie et al. 2008), Enicocephalomorpha + Dipsocoromorpha (Wang et al. 2016), or these two infraorders + Gerromorpha (Johnson et al. 2018) as a sister group to the remaining infraorders, or Panheteroptera. In all studied species of Peloridiidae (Coleorrhyncha), which is usually considered as a sister group to Heteroptera both on morphological (Schlee 1969; Grimaldi and Engel 2005), molecular (Xie et al. 2008; Cryan and Urban 2012), and cytogenetic (Kuznetsova et al. 2015) grounds, testes also consist of a single follicle (Grozeva et al. 2014).

Reconstruction of an ancestral state for ovaries (Fig. 3) indicates a similar, but more straightforward, result, giving seven ovarioles p.o. for the entire clade, Pentatomomorpha (and all recognized superfamilies except Aradoidea), Cimicomorpha (and all included superfamilies except Microphysoidea and Reduvioidea), Leptopodomorpha, Gerromorpha, and Nepomorpha. The infraorders Enicocephalomorpha and Dipsocorimorpha form an exception, with five ovarioles p.o. recovered for each of them as an ancestral state.

Summing up, the number of seven appears to be an ancestral state for both testes and ovaries of true bugs. It is still retained in many lineages but may increase or decrease in separate groups. The trend towards decreasing the number of follicles and ovarioles undoubtedly prevailed in the evolution of the suborder Heteroptera in general.

## Supplementary materials

Supplementary material 1 An updated list of testicular follicles’ and ovarioles’ numbers in Heteroptera Data type Morphological data https://binary.pensoft.net/file/783441This dataset is made available under the Open Database License (http://opendatacommons.org/licenses/odbl/1.0/). The Open Database License (ODbL) is a license agreement intended to allow users to freely share, modify, and use this Dataset while maintaining this same freedom for others, provided that the original source and author(s) are credited.Snejana Grozeva, Desislava Stoianova, Fedor Konstantinov, Nikolay Simov, Valentina G. Kuznetsova
